# Intravenous Thrombolysis in Ischemic Stroke Patients With Active Cancer

**DOI:** 10.3389/fneur.2018.00811

**Published:** 2018-09-28

**Authors:** Henriette Aurora Selvik, Halvor Naess, Christopher Elnan Kvistad

**Affiliations:** ^1^Department of Clinical Medicine, University of Bergen, Bergen, Norway; ^2^Department of Neurology, Haukeland University Hospital, Bergen, Norway; ^3^Centre for Age-Related Medicine, Stavanger University Hospital, Stavanger, Norway

**Keywords:** ischemic stroke, cancer, thrombolysis, adverse events, stroke treatment, hemorrhage

## Abstract

**Introduction:** It has been difficult to state specific guidelines for IV-tPA use in cancer patients. Many of the randomized tPA-trials included too few patients with cancer or excluded patients with cancer entirely. In this report, we aimed to study the use of IV-tPA in patients with active cancer and acute ischemic stroke. We also investigated if the cancer patients who received IV-tPA experienced adverse events.

**Methods:** All patients with ischemic stroke admitted to the Stroke Unit at Haukeland University Hospital were prospectively registered in the NORSTROKE database and every patient's medical record was searched for cancer diagnoses.

**Results:** Of 1,646 patients admitted with ischemic stroke, 82 (5.0%) patients had active cancer. The total number of patients treated with IV-tPA was 16.2%. Five patients with stroke and active cancer were treated with IV-tPA (6.1%) and none suffered adverse events. Of the patients with no history of cancer, 261 (16.7%) were treated with IV-tPA and 3.8% experienced tPA-related adverse events.

**Conclusions:** Few patients with active cancer receive thrombolysis for acute ischemic stroke. We report five cancer patients (three known and two occult) treated with IV-tPA for ischemic stroke without tPA-related adverse events.

## Introduction

Cancer can induce hypercoagulability, leading to both venous and arterial thromboembolism (1). The prevalence of active cancer in ischemic stroke patients is around 5% (2). However, this may be an underestimation as autopsy evidence has shown that up to 15% of cancer patients undergo cerebrovascular ischemia, of which up to half remain undiagnosed. Given this finding, it was suggested that cancer patients suffer milder strokes than other patients, which thus remain undiagnosed and untreated (3). If a patient is suffering an acute ischemic stroke, the pillar of stroke treatment is intravenous (IV) thrombolysis (tPA). Guidelines for treating cancer patients with IV-tPA are still unclear (4). As cancer-associated stroke gains more attention, the new American Heart Association/American Stroke Association guidelines state the high hemorrhage risk of IV-tPA treatment in patients with structural gastrointestinal malignancies and cancer patients post-operatively (5). However, due to lack of substantial data, it has been difficult to state general guidelines for IV-tPA use in cancer patients because many of the randomized IV-tPA-trials included too few patients (6) or excluded patients with cancer (7).

In this report, we aimed to study the use of IV-tPA in patients with active cancer and acute ischemic stroke. We compared IV-tPA treated patients with active cancer to IV-tPA treated patients with no history of cancer. We also investigated if the cancer patients who received IV-tPA experienced any tPA-related adverse events.

## Materials and methods

Inclusion of patients started in January 2006 and ended in September 2012. All patients with ischemic stroke admitted to the Stroke Unit at Haukeland University Hospital were registered in the NORSTROKE database, a comprehensive research registry, and every patient's medical record was searched for cancer diagnoses. Ischemic stroke was defined in accordance to the Baltimore-Washington Cooperative Young Stroke Study Criteria as neurological deficit lasting more than 24 h or transient ischemic attacks (TIA) where computed tomography (CT) or magnetic resonance imaging (MRI) showed infarctions related to the clinical findings (8). Stroke severity was determined by the National Institutes of Health Stroke Scale (NIHSS) on admission. Stroke etiology was determined by sing the Trial of Org 10172 in Acute Stroke Treatment (TOAST) criteria (9). Active cancer was defined as (1) cancer diagnosis, (2) metastasis of known cancer, (3) recurrent cancer or (4) receiving cancer treatment within 12 months *before or after* the index stroke. Patients were dichotomized as patients with known active cancer (CS) and no evidence of active cancer (NCS). Adverse events were defined as any unwanted side effect from the IV-tPA, including intracerebral hemorrhage (ICH).

## Results

Of 1,646 patients admitted with ischemic stroke, 82 (5.0%) patients were diagnosed with cancer within 12 months prior to or post-stroke ictus (CS), of whom 46 were diagnosed pre stroke and 36 were diagnosed post-stroke. Thus, 1,564 stroke patients had no history of cancer for the duration of the study.

Of all stroke patients, 266 patients were treated with IV-tPA upon admission (16.2%). Five of the CS patients were treated with IV-tPA (6.1%), while 261 (16.7%) of NCS patients were treated with IV-tPA for their acute ischemic stroke. Of the IV-tPA-treated CS, three were diagnosed pre stroke, while two CS were diagnosed shortly post-stroke and thus had occult cancer at stroke ictus.

Table 1 shows the characteristics of the IV-tPA-treated CS patients vs. IV-tPA-treated NCS patients. CS patients more often suffered multiple acute cerebral infarcts (MACI), had a higher mean glucose on admission and a lower hemoglobin level. Platelet count and other blood test did not differ between CS and NCS patients (Table 1).

**Table 1:** Characteristics of cancer patients who received IV-tPA vs. never cancer who received IV-tPA.

	**CS & IV-tPA (*n* = 5)**	**NCS & IV-tPA (*n* = 261)**	***P***
Male, *n* (%)	4 (80.0)	164 (62.8)	0.4
Female	1 (20.0)	97 (37.2)	
Time from ictus to admission	73.1 (45.0–123.8)	90 (59.1–135.0)	0.5
Mean age (SD)	68.0 (10.4)	68.7 (14.9)	0.9
Median NIHSS, arrival (IQR)	9 (8–14)	7 (4–15)	0.9
Median NIHSS, discharge (IQR)	11 (0–19)	3 (0–9)	0.4
ICH; tPA complicaiton	–	10 (3.8)	0.7
Median mRS (IQR)	4 (2–5)	2 (1–4)	0.4
Persistant AF	1 (10.0)	9 (3.4)	0.06
Paroxysmal AF	1 (10.0)	21 (8.0)	0.3
Smoking	4 (80.0)	137 (52.5)	0.08
MACI	2 (40.0)	14 (5.4)	0.001
**STROKE ETIOLOGY**			
Atherosclerosis, *n* (%)	–	40 (15.3)	0.6
Cardioembolic	2 (40.0)	91 (34.9)	
Small-vessel disease	1 (10.0)	14 (5.4)	
Other	–	10 (3.8)	
Unknown	2 (40.0)	105 (40.2)	
**BLOOD VALUES**			
Median D-dimer (IQR), mg/L	0.6 (0.3–2.8)	0.7 (0.4–1.6)	0.9
Mean Hb (SD), g/dL	12.5 (2.1)	14.4 (1.4)	0.002
Mean platelet count (SD), × 10^9^/L	292.0 (104.7)	265.9 (78.5)	0.5
Median fibrinogen (IQR), mmol/L	3.1 (2.8–4.1)	3.4 (3.0–3.9)	0.4
Median CRP (IQR), mg/L	1 (1–6)	3 (1–6)	0.3
Mean cholesterol (SD), mmol/L	4.1 (1.1)	5.4 (1.2)	0.07
Median glucose (IQR), mmol/L	8.0 (7.1–8.3)	6.2 (5.4–7.3)	0.02

*SD, standard deviation; NIHSS, National Institutes of Health stroke Scale score; IQR, interquartile range; mRS, modified Rankin score; AF, atrial fibrillation; MI, myocardial infarction; PAD, peripheral artery disease; and MACI, multiple acute cerebral infarctions*.

Time from ictus to admission was somewhat shorter for IV-tPA-treated CS patients, but not significantly so [73.1 min (IQR 45.0–123.8) vs. 90 min (IQR 59.1–135.0), *p* = 0.5]. Equally, for the patients who did not receive IV-tPA (*n* = 1,378), the time from stroke ictus to admission was similar between CS- and NCS patients [289.7 min (IQR 123.8–1,020.9) vs. 353.0 (IQR 129.4–1,181.3), *p* = 0.8].

Fifteen CS patients (19.2%) and 277 (21.3%) NCS patients arrived at the hospital within 270 min and were as such eligible for IV-tPA, yet did not receive IV-tPA for varying reasons and contraindications.

Table 2 presents the five CS patients who received IV-tPA in more detail. Three had colon cancer (1 of whom was occult), and the remaining two had malignant melanoma (1 of whom was occult). Two had a stroke etiology of cardioembolism, one small-vessel disease and two undetermined etiology.

**Table 2:** Characteristics of the five patients with cancer who received IV-tPA.

	**Age, sex**	**Cancer type**	**Cancer treatment if prior to stroke**	**Cancer diagnosis, date**	**Stroke, date**	**NIHSS on admission**	**NIHSS on discharge**	**Time: ictus to tPA**	**tPA adverse event**	**Note**
Patient 1: Known cancer	52, male	Colon cancer, lymphnode metastasis	Surgery Oct. 6th 2006. Chemother. from Nov. 17th 2006: (Oxaliplatin + Fluoruracil + Kalsiumfolinat) + Avastin (Bevacizumab)	Sept. 6th 2006	Dec. 8th 2006	**13**	**11**	1 h 15 min.	None	Recurrent stroke 2 days later; treated with heparin, no IV-tPA for recurrent stroke.
Patient 2: Occult cancer	77, female	Colon cancer	No treatment pre stroke	Dec. 17th 2007	Dec. 2nd 2007	**15**	**23**	2 h 10 min	None	Warfarin seponated 2 weeks prior to stroke due to rectal hemorrhage.
Patient 3: Known cancer	69, male	Malignant melanoma	Surgical resection, July 2010.	July 21st 2010	Nov. 8th 2010	**14**	**19**	2 h 31 min.	None	–
Patient 4: Known cancer	62, male	Malignant melanoma	No treatment pre stroke.	Nov. 14th 2010	April 17th 2011	**8**	**0**	3 h	None	Died from cancer shortly after stroke. Widespread metastasis.
Patient 5: Occult cancer	77 male	Colon cancer	No treatment pre stroke.	Aug. 29th 2011	July 20th 2011	**9**	**0**	1 h 15 min	None	Bladder cancer in 2003. Treated surgically & BCG inj.; full remission.

*Bold values represent the NIHSS score as determined by stroke physician, on admission (pre tPA) and on discharge (post tPA)*.

Only one of the patients was treated with chemotherapy prior to stroke. None of the five IV-tPA-CS patients experienced ICH or any other adverse event from the IV-tPA treatment.

## Discussion

In our cohort, ~16% of NCS patients were treated with IV-tPA for their acute ischemic stroke. Meanwhile, of patients with active cancer, only about 6% of the CS patients were treated with IV-tPA. The largest report to date on thrombolysis in cancer patients with acute stroke, had yet a lower IV-tPA-rate of 1.6% for CS patients, and 2.9% for NCS patients (7). In the present study, two of the five CS patients had occult gastrointestinal cancer, specifically colon cancer. If they would have been IV-tPA treated if this was diagnosed is therefore unknown.

As postulated by Graus et al., we wondered if fewer patients with cancer received IV-tPA due to less focal and milder stroke symptoms (3). On the contrary, and as comparable to other pre-mortem studies, we found that the cancer patients who suffer stroke and cancer experience equally or more severe strokes (10).

It is not inconceivable that some cancer patients do not receive IV-tPA due to the treating physician's uncertainty. This could be due to both the inherent fear of adverse events and hemorrhage, but possibly also the belief that because of significant CS patient co-morbidity, IV-tPA treatment ultimately is futile in bettering the outcome. As such, the willingness to risk tPA-related hemorrhage could diminish further. Cancer is not an established contraindication for IV-tPA, yet, due to lack of empirical evidence of the safety of IV-tPA in cancer patients, physicians have been hesitant to use it. Some cancer patients have coagulation disorders, some have thrombocytopenia, a relative contraindication, or even non-bacterial endocarditis, associated with hemorrhage (11, 12). Although none of the present study's CS patients had thrombocytopenia, this might have been seen if the number of CS patients was higher.

The most important question remains; is it safe to use thrombolysis in patients with active cancer? In our report, we present five patients with active cancer who received IV-tPA, of whom none experienced adverse events. As mentioned, the largest study on this topic with >800 CS patients also concluded that it was safe. However, this study used administrative data and International Classification of Disease codes for their analyses and may have missed significant patient data (9). The most recent paper studying thrombolysis treatment in acute stroke patients with cancer argues that cancer patients more often experience hemorrhagic transformation and poorer outcomes (6, 10). It is not, however, unexpected that patients with comorbid stroke and cancer have increased mortality and poorer outcomes post-stroke (1). As such, it may not be warranted to conclude that IV-tPA is unsafe based on a study of 12 patients, especially as the larger studies suggest that IV-tPA is in fact equally safe in patients with cancer. It is noteworthy that cancer patients form an immensely heterogeneous group, and it may therefore be difficult to create a thrombolysis guideline for the group as a whole. For instance, cases have shown that IV-tPA is unsafe for patients with intraaxial brain tumors, yet safe for those with extraaxial brain tumors (13).

A limitation to this report is the low number of patients included. The small study population thus leaves us with lack of power to compare the findings to other studies. Higher numbers of patients and varying cancer types and data on cancer stage and treatment is needed to create guidelines for treating cancer patients with IV-tPA.

## Conclusions

Few patients with active cancer receive thrombolysis for acute stroke. The literature indicates that IV-tPA treatment is equally safe in patients with stroke and cancer. In accordance, we report five cancer patients (three known and two occult) treated with IV-tPA for ischemic stroke without tPA-related adverse events.

## Ethics statement

Informed consent, or consent from legal guardian, was obtained from all patients. The study was approved by the Regional Committee for medical and health research ethics (REK Vest).

## Author contributions

HS is the main author of this report, she has performed the statistical analyses and written the first draft of the paper. She has contributed in all parts of the paper-writing process, as well as contributed HN in data collection. CK has contributed to the main hypothesis and idea of the paper, and he has supervised its process until completion. He also had the final read-through. HN is the one who founded the research registry used, and is thus responsible for data collection and patient data. He has also contributed in the writing of the manuscript.

### Conflict of interest statement

The authors declare that the research was conducted in the absence of any commercial or financial relationships that could be construed as a potential conflict of interest.
